# On the Prevention of Crime
*An address delivered to the grand jury.


**Published:** 1852-01-01

**Authors:** M. D. Hill

**Affiliations:** RECORDER OF BIRMINGHAM


### ON THE PREVENTION OF CRIME.*

BY MR. M. D. HILL, Q.C., RECORDER OF BIRMINGHAM.

GENTLEMEN OF THE GRAND JURY,—At the Michaelmas sessions of last year, I addressed your predecessors on the repression of crime, confining, however, my remarks to one branch of that great subject—namely, the propriety of holding in restraint known malefactors, who could be shown on sufficient evidence to pursue crime as a calling, although by their dexterity and good fortune they had been able to elude the proof of any specific offence. This charge drew public attention to an extent for which I was not prepared. By those best acquainted with the class to be held in check, and with their manifold inflictions on society, I believe I may venture to state that it met with acceptance; but having been handled by men of acute minds, unguarded from error by practical experience, I ought not to wonder that a new question, or, at all events, a question new to

* An address delivered to the grand jury.

these critics, would call forth dissent as well as agreement—dissent exhibiting itself in a multitude of ingenious objections. If I had foreseen that any observations falling from me could have been deemed worthy of so much notice, I might have thought it prudent to offer my views to the public under circumstances which would have enabled me, by treating the subject in greater fulness than can well be done in a charge, to have answered by anticipation the objections which have been urged against me. My reply I intend to give on the present occasion, and, as I hope, without drawing too much on your patience. No doubt I might have taken an earlier opportunity of performing this task; but I thought it due to the diversity of opinion to which I have adverted to take ample time for a consideration of what I had submitted to your predecessors, in order that by a careful and (so far as any efforts of mine could insure it) a candid review of all that has been urged on the one side and the other, I might either maintain my position or retract my errors, and give at the same time publicity to the reasons which had led to my change of opinion. And, gentlemen, if I know myself, I should not have felt humiliated by such a retractation. On the contrary, it would have been satisfactory to me to reflect that the discussion which I had originated had proved the fallacy of a remedy which, having been plausible enough to mislead one searcher after truth, might decoy others of more power and influence, and thus lead to its being carried into action. This review I have at length made, and have weighed the arguments on both sides. I have also taken into account some general facts, which have either come into existence in the interval, or have been made more prominent than before; and I am bound to avow myself confirmed in my original views. Gentlemen, I submitted to your predecessors a speculative opinion and a practical proposal. My speculative opinion was, that all persons living without visible means of support, and who, in the belief of witnesses acquainted with their way of life, are maintaining themselves by crime as their stated calling, ought to be called upon to prove themselves in the enjoyment of some honest means of subsistence; and I further submitted that, in the absence of such proof, they should be bound to give sureties for good conduct; and again, that failing to give satisfactory security, they should be committed to prison for a limited period. This was my theory. And it was founded on the well known fact (which I pause for a moment to state has never yet been controverted), that each individual of the class of professional marauders is well known, both personally and by character, to the police and to his neighbours, and could be pointed out with perfect ease. From this fact I drew the consequence that society (having such means of knowledge within its reach) was not only justified, but bound to use it for the general protection. In my practical proposal, however, I stopped short, and limited the application of my theory to the cases of offenders who had already been convicted. I adopted this limitation for several reasons; one, that it is always well to proceed step by step in an untried course, or in a course comparatively untried; *another*, because convicted criminals form a large, and by far the most dangerous portion of the predatory class; and thirdly, because by conviction they have necessarily forfeited the confidence of society. That they have been guilty men is an established fact, while in the majority of instances there is neither evidence nor probability of their having abandoned their evil courses. Indeed, how should there be? The administration of the law proceeds on the principle of retribution. The criminal is convicted of a given offence, and has measured out to him a given length of punishment. It is true that, during his term of confinement, we take some steps to reform him, which are more or less adapted to attain that end. But his detention is neither in the first instance regulated by an estimate of the time required for that purpose, nor is there any power to continue it until his reformation is effected. The prisoner is afflicted with a moral disease, but the prison cannot be considered in the light of a hospital for its treatment without exposing the administration of criminal justice to ridicule. For what should we think of a hospital for the cure of a malignant and infectious disease (and surely no disease can be more malignant or more infectious than crime), if the rule of its governors were to keep the patient, not until he is cured, but a week, a month, or a year, according to a principle of regulation quite irrespective of his condition at the time of his dismissal, and making it altogether a matter of accident whether he is relieved of his distemper, or whether he is sent forth to spread infection through the land.

As long, then, as punishment is measured out upon the retributive principle, so long an individual once convicted must remain an object of just and unavoidable suspicion, and the class to which he belongs may reasonably be selected for any experiment which the welfare of the community requires to be instituted. To those who have made it a topic of observation and inquiry, it is well known that criminals not unfrequently pursue a system of depredation with impunity for long periods. With regard to one man very lately sentenced to transportation, it has from peculiar circumstances occurred to me to know, that his career of crime has extended over more than thirty years without a single conviction; and I have strong reasons for believing that his is by no means a solitary case. Almost every newspaper contains some paragraph narrating a criminal exploit in which there is a combination of skill and boldness, marking out the perpetrator as experienced in the violation of the law. We often read of attacks in streets and other frequented thoroughfares, by ruffians who seem to have taken as their model the Indian Thug; and their feats prove them to be as dexterous as their masters, while in audacity they leave him far behind. Such outrages as these, gentlemen, are not the acts of tyros in villany. They imply the skill, the contempt of danger, and indifference to the sufferings of their victims, which training, and training alone, can give. And here we cannot shut our eyes to the fact, that our present system of punishment offers great facilities, not to say inducements, to a training to crime. In order to place this unhappy tendency in a clear light, let me suppose for a moment that it were the object of society to defeat the intention of its own laws, and to strengthen the propensity to crime in every individual in whom such propensity had ever been disclosed by the commission of an offence. Let us compare our present mode of proceeding, as to criminals, with that which we pursue when our wish is not to deter, but to stimulate and encourage. And I think you will observe a wonderful similarity. What is our treatment of our children in their education? Do we not give them short and easy lessons at first, lest they should be disgusted with learning at the outset, and so close their minds against the lessons of their teachers? And do we not augment their tasks with the growth of their strength, and in proportion as practice adds to their ability for mental application? Do we not, in short, graduate the rate of their progress according to their powers of action and endurance? Well, then, let us now consider our treatment of criminals. When the juvenile offender first presents himself at the bar we give him a slight imprisonment, just enough to accustom him to short separations from his companions, and to dispel the wholesome illusion which had made the gaol a place of fear, because it was a place of mystery. On the next occasion he remains longer; but he has become practised in prison life, and bears confinement far better than he would have done but for his former lesson. This process is repeated from time to time, while the moral which the wretched creature draws from his alternations of confinement and freedom is, not to refrain from offending, but to commit offences in such a manner as shall least expose him to the risk of detection; and, moreover, that when at length detected he ought to bear his privations with as much of contempt and defiance as he can command—consoled by the prospect of restored freedom and the hope of better fortune in future. Is not this, gentlemen, a fair parallel? And does it not show that our treatment of malefactors is better calculated to confirm them in evil doing than to withdraw them from crime? It will be observed that I speak of the general working of our system. That there are many exceptions to the rule I am glad to believe. No man can appreciate more highly than I do the labours of many governors and many chaplains—aided, as they are often, by volunteers of both sexes, who look on the criminal, not as an outcast to be flung aside in contempt and hatred, but as an erring brother, to be reclaimed from guilt, if by the most strenuous and persevering efforts of well directed kindness that great end can be reached. If then, gentlemen, the foregoing remarks are well founded, the number of convicted malefactors roaming at large must excite much less of surprise than alarm; but I greatly fear that we are yet to expect considerable additions to this body. It is well known to you, as to all persons of education, that during the last forty years (dating from the time of that great and good man, Sir Samuel Romilly) there has been a steady progress made by the legislature in mitigating the severity of our criminal code, which, when he began his labours, was the most sanguinary to be found in the civilized world. Neither can it have escaped your observations, that the sentiment which has actuated the legislature has also prevailed in the administration of criminal justice. Indeed, society through all its gradations is imbued with a far milder spirit than in bygone times. The combined operation of these causes has been not only to shorten terms of imprisonment, but to make the severer penalty of transportation of less frequent occurrence in proportion to the number of convicts than heretofore—a circumstance which would have attracted more attention if the difficulty of ascertaining the numbers actually sent out of the country at different periods were less than it is and always has been. And now an additional obstacle in the way of transportation has arisen, which threatens very seriously to lessen, if not altogether to extinguish this kind of punishment. Penal colonies, planted by the mother country at a vast expense for the disposal of her convict population, and which formerly were the willing recipients of these degraded persons (gladly availing themselves of the ample supply of labour thus afforded for bringing their tracts of new land into cultivation) have at length discovered, that the moral evils incident on the importation of malefactors far outweigh the material benefits to which they (the colonists), had hitherto limited their calculations. It would be unbecoming in me, while sitting here, to enter into the controversies and heartburnings which have arisen out of this change in colonial policy. All that I desire at the present moment is to call your attention to the portentous consequences which may, and as I think must, result from the impediments thus thrown in the way of transportation, when taken in connexion with the causes to which I have adverted, as lessening the numbers on whom that punishment would be inflicted, even if the facilities for carrying it into effect were as great as they continued to be up to a recent period. This consequence is, the permanent augmentation around us in the number of liberated convicts. What that addition will amount to it is of course impossible to predict, but that it must be very large is pretty certain, from the experience of countries having no colonial outlets, and because, although sentences for transportation were at all times more frequently for limited terms of years than for life, so few returned, that the country might almost be said to be freed for ever from the presence of a convict when once he had left our shores.

Do not, gentlemen, mistake me by imagining that I am pronouncing an eulogy on transportation. Believe me, I bear too clearly in my mind the powerful and conclusive arguments by which it has been assailed. All I desire to impress on you is, that the stoppage of that great sewer which has for so many years carried away the dregs of our population, will produce a most unwholesome effect, other things remaining as they are; and that while the country adheres to the principle of retributive punishment (as it probably will do long after the voice to which you so kindly listen is hushed in the grave), so long that pernicious effect will imperatively call for some special remedy—which remark brings me back to the consideration of the one laid before your predecessors, and of which I have already given you a sketch. This I will now more fully describe. I propose that every person who has been convicted of felony, or of a misdemeanour implying fraud (as obtaining goods under false pretences, knowingly passing base coin, and the like), shall be liable to be dealt with as follows:—If, after the expiration of his imprisonment under his conviction, he shall be brought before a magistrate, charged with still persevering in crime, it shall be the duty of the magistrate, if the witnesses by evidence of general conduct satisfy his mind that the charge is established, to call on the prisoner to show that he enjoys the means of honest subsistence either from his property, his labour, the kindness of his friends, the bounty of the charitable, or from his parish. Should he succeed in adducing this proof he is to be discharged. Should no such proof be forthcoming, he is next to be called upon to give bail for his good behaviour. Supposing him to answer this demand, he is to be still entitled to his discharge. But in the event of his failure, he is then to be held to bail on his own recognizances, and his case is to be sent to a jury at the assizes or sessions, when, if a verdict pass against him, he is to be imprisoned for a term to be fixed by the law, but capable of diminution by the judge before whom he is tried. This, gentlemen, is my proposal in detail, and, perhaps, it will appear to you, as it did to your predecessors (who honoured it with their approval when I submitted it to them in outline), that it sufficiently guards the accused against the danger of being deprived of his liberty on fallacious grounds. In the first place, no proceedings under the proposed law would put the convict into custody even for a day, except by the verdict of a jury; unless, indeed, he should forfeit his recognizances by not appearing to take his trial, when he would subject himself to the well-known consequences of such a contempt. Suppose him, then, on his trial, and observe how he is fenced round with protections, "covered," as Erskine expresses it, "from head to foot with the panoply of the law." In the first place, his accusers must satisfy the jury that he was at the time of his apprehension in the course of life which they charge upon him, not merely that he was so before his conviction. This evidence he will rebut, if he can, either by impeaching the character of the witnesses, showing that their statements are false or inconclusive, or by explaining away the facts established against him. And in this part of his case, as in all other parts, he may adduce witnesses of his own. But suppose him to fail in meeting the charge, he then falls back on his second defence, and shows the manner in which he subsists. Now, if he have in truth an honest income, it is not very easy even to imagine a set of circumstances which disable him from proving a fact so emphatically within his own knowledge. But we will go on to suppose him defeated in this second defence. Even then, unless he is altogether bereft of honest friends, having confidence that he will not commit crime, he finds bail and remains at liberty.

Now, gentlemen, the species of objection to which I thought my proposal most obnoxious is, that it offers too many chances of escape to be practically efficient for the restraint of criminals. On this head, however, none who are conversant with the life and habits of the class in question have the least misgiving, nor has that objection ever been advanced. On the contrary, the numerous attacks which the plan has undergone have been always directed against the danger of committing injustice on the convict. That such a miscarriage is within the limits of possibility I must admit, but that such trials as I propose are more open to this reproach than trials for specific offences, or so open, I do take upon myself, speaking from a very long experience in criminal courts, confidently to deny. No tribunal is infallible. No discovery has yet been made which supplies a sure touchstone to human testimony. And if the lamentable fact that innocent men are sometimes convicted were sufficient for the condemnation of criminal jurisprudence, no mode of trial that the wit of man has ever invented could stand. But from the strain in which some writers have indulged, it might be supposed, if experience had not recorded a very different result, that trials for specific offences never failed of bringing out the truth, always acquitting the innocent, and ascertaining with exactitude the criminality of the guilty. One short statement will dispose of this fond belief, if any person is so misled as to entertain it. The brother of the Lord Chancellor, Mr. Edward Wilde, was the benevolent instrument, during his year of office as Sheriff of London, of saving six persons from death, showing on one occasion that the prisoner was clearly innocent; on others, that conclusions had been hastily drawn from facts which did not justify them, and thus nullifying the proofs of guilt; and with regard to the remainder, adducing evidence which went so far to mitigate their conduct, as to prove that to put them to death would be a most unjustifiable measure of severity. Whoever to whom these events are known as they were to me from time to time as they occurred—whoever reflects that they happened in one court and in one year (nay, in less than one year, for Mr. Wilde held office only for ten months), must see that confining the charge to one specific transaction by no means ensures success in the attainment of the truth. One source of miscarriage is, indeed, peculiar to such trials, and that happens to be the most frequent by which the administration of justice is beset. I allude to mistakes as to the identity of the prisoner with the party really guilty. Misconceptions of this kind belong only to moments of time (or at all events to very short periods), and cannot occur when the question relates to general conduct and the tenour of a man's life. Moreover, when a specific offence is charged, it is no conclusive answer (nor can it be) that the prisoner had means of livelihood, and therefore is not to be supposed guilty, which in the prosecutions suggested by me it is always competent to furnish. And now let me, gentlemen, ask a plain question. Is a man who has already been convicted, whose conduct is such that a jury is satisfied he is still a malefactor, who, being then called on to explain how he obtains his livelihood, has no answer to give, who is so distrusted by all the world that he cannot find bail for his good conduct; is that man—that pest of society—to remain at large? Ought we, on the mere surmise that errors may creep into the trial of such persons (in spite of all the care which has been taken to exclude them), to hold back from the exercise of a jurisdiction of admitted potency for the attainment of its object, when that object is clearly of such vital importance? Gentlemen, the crying necessity of this jurisdiction so presses itself on my mind that I cannot refrain from adverting to it once more. But few days have elapsed since the part of England in which I reside (the county of Somerset) was the scene of an appalling outrage, filling the district with indignation and horror. A girl, fifteen years old, was left by her parents alone at their dwelling, during the necessary attendance at the neighbouring market of Frome. On their return home, they found her dead body stretched on the floor and dabbled in blood. In the open day—in a house not distant from others of the hamlet, and near to a main road —had this unhappy girl lost her life in the defence, and, alas! in the unsuccessful defence, of her purity! The pangs of death were sharpened by the cruel ignominy of violation. How much less hideous had been her fate—how much less bitter the grief of her bereaved parents, had she been devoured by a beast of prey! Her image would then have dwelt in their memory unsullied by those revolting associations of pollution with which it will now for ever be mingled. Is the convict, then, I ask, to exhaust all our sympathies?

Are we to have no thought for the myriads of honest and faithful subjects exposed to the same frightful perils, deeply feeling the want of protection, the comfort of whose lives is ofttimes destroyed by the perpetual fear which harasses their minds? But, gentlemen, we almost always find that an over-wrought strictness in one direction is balanced by some glaring laxity in another. Writers who evince the greatest trepidation at the proposal to which your attention has been drawn, themselves urge the adoption of an alternative infinitely more perilous to innocence than the most distorted imagination can figure to itself out of mine. Deliberate advice has been given, that each man should defend his dwelling with fire-arms. Let us pause for a moment to examine what this advice implies. It implies that a person suddenly aroused from sleep, in the dead of night, and in all the disturbance of mind which an impending conflict must produce, is, while pointing his blunderbuss and drawing the trigger, to accuse, try, and condemn a suspected burglar, discerned for an instant in the dark, and to execute upon him the irrevocable doom of a capital punishment. Surely, for such very fastidious legislators, this is a somewhat startling recommendation. But what has resulted from the promulgation of this advice? Gentlemen, within a very short interval of time, two innocent persons, one of them an officer of police—a protector, instead of an assailant—have fallen, by the hands of clergymen too, who (as we should all agree), if the power could be safely exercised by any class of the community, are best entitled to the trust, by the self-restraint and the merciful spirit which pertain to their sacred calling, and by the reluctance which, above all others, they must feel at sending a fellow-creature to his account with all his sins upon his head. Nevertheless, gentlemen, if the law will permit known ruffians to remain at large, these barbarous remedies, perhaps, cannot, and most certainly will not, be dispensed with; yet, who does not see that any method of trial, however rude and defective, even Lynch-law itself, is infinitely to be preferred? I have now, gentlemen, I trust, shown that my plan is not open to the objections which have been raised against it; but I cannot conclude without (paradoxical as it may appear) avowing that I am far more gratified than disconcerted at these objections. They prove how deeply Englishmen are imbued with instinctive reverence for the liberty of the subject. This, like every other sentiment, may be carried to an unwarranted length. On the question before you I think it has been so treated; but I for one will ever bear in mind that personal freedom is the surest foundation of our other liberties, and that hostility to any interference with it challenges my respect, even when it exceeds the limits of a reasonable jealousy. If, then, on calm consideration, my proposal shall be found, by the verdict of reflective men, unwisely to infringe on that noble privilege, none will rejoice more sincerely than myself that I have not been taken at my word. Grateful shall I be to those who will have saved me from the life-long sorrow of having inflicted injury where I had humbly hoped to suggest an important benefit. Thanks, gentlemen, for your patience—your task is finished.

---

